# Assessment of the Functional Regions of the Superantigen Staphylococcal Enterotoxin B

**DOI:** 10.3390/toxins5101859

**Published:** 2013-10-22

**Authors:** Lily Zhang, Thomas J. Rogers

**Affiliations:** Center for Inflammation, Translational and Clinical Lung Research, Temple University School of Medicine, 3500 N. Broad Street, Philadelphia, PA 19140, USA; E-Mail: lzhang@temple.edu

**Keywords:** superantigen, staphylococcal enterotoxin B, MHC class II, T cell receptor

## Abstract

The functional activity of superantigens is based on capacity of these microbial proteins to bind to both the β-chain of the T cell receptor (TcR) and the major histocompatibility complex (MHC) class II dimer. We have previously shown that a subset of the bacterial superantigens also binds to a membrane protein, designated p85, which is expressed by renal epithelial cells. This binding activity is a property of SEB, SEC1, 2 and 3, but not SEA, SED, SEE or TSST. The crystal structure of the tri-molecular complex of the superantigen staphylococcal enterotoxin B (SEB) with both the TcR and class II has previously been reported. However, the relative contributions of regions of the superantigen to the overall functional activity of this superantigen remain undefined. In an effort to better define the molecular basis for the interaction of SEB with the TcR β-chain, we report studies here which show the comparative contributions of amino- and carboxy-terminal regions in the superantigen activity of SEB. Recombinant fusion proteins composed of bacterial maltose-binding protein linked to either full-length or truncated toxins in which the 81 N-terminal, or 19 or 34 C-terminal amino acids were deleted, were generated for these studies. This approach provides a determination of the relative strength of the functional activity of the various regions of the superantigen protein.

## 1. Introduction

The staphylococcal enterotoxins are members of a family of gram-positive pyrogenic exotoxins possessing superantigen activity. The staphylococcal enterotoxins, toxic syndrome shock toxin-1 (TSST-1), and streptococcal pyrogenic exotoxins are structurally related proteins [1,2,3]. These bacterial superantigens possess two common properties. First, they bind with moderate affinity to major histocompatibility complex (MHC) class II dimers [4,5], and second, these toxins are recognized by the T cell receptor (TcR) in a β-chain variable region allele-selective fashion [6,7]. It is now understood that the bacterial superantigens possess additional functional activities which are likely to involve other binding sites. For example, emetic activity which is dependent on the disulfide loop [8], and at least two binding sites which have been identified that are involved in epithelial cell binding activity [9,10].

The structural basis for the superantigen activity of these toxins has been the subject of intense research. Several approaches have been used to determine the regions of the bacterial superantigens involved in the mitogenic activity of these toxins. These include the use of synthetic peptides corresponding to regions of staphylococcal enterotoxin A (SEA) to block the activity of the native toxin [11,12], the analysis of the activity of proteolytic digestion fragments of several of the toxins including SEA, SEB, SEC1, SEC2, and TSST-1 [13,14,15,16,17,18,19], the use of monoclonal antibodies specific for identified epitopes to neutralize superantigen activity [16,17,18], the characterization of recombinant mutant SEA, SEB, TSST-1, and streptococcal pyrogenic exotoxin A (SPEA) containing amino acid substitutions [20,21,22,23,24], and the analysis of toxin chimeras to localize regions involved in the TcR Vβ allele selectivity [25,26]. Results from experiments carried out to determine the location of epitopes responsible for the biological activities of the superantigens have frequently appeared contradictory. 

Several of the bacterial superantigens have now been crystallized [27,28,29,30], and it has been suggested that the staphylococcal and streptococcal superantigens can be grouped evolutionarily [31,32]. Analysis of the SEB crystal structure indicates that this protein consists of two domains with predominant β sheet structure, and the amino- and carboxy-termini of SEB are in close proximity. The crystal structure data of the SEB/class II complex [33], and the complex of SEB with the TcR [34,35,36] have provided evidence that the both amino- and carboxy-terminal residues of SEB may participate in interaction with both the MHC class II and the TcR. However, the relative contributions of residues in these regions to the binding interactions of these toxins have not been entirely clarified.

An additional approach utilized to identify regions of the toxins which contribute to superantigen activity involves the generation of recombinant truncations or internal deletions [37,38,39,40,41]. Hedlund *et al*. [37] have reported results showing that an N-terminal 106-amino acid SEA truncation possesses normal MHC class II-binding activity, suggesting that the region 107–233 possesses the epitopes strongly involved in this function. On the other hand, additional work [41] has shown that deletion of as few as 60 N-terminal amino acids results in greatly impaired SEB activity. It is clear, however, from the solution of the crystal structures of SEB [27] that the C-terminus of these bacterial superantigens folds back onto the N-terminal residues. 

One limitation in the use of a superantigen with altered structure is that the mutation may lead to unexpected conformational changes, or decreased stability. Several investigators have employed the fusion protein approach in an effort to stabilize the structurally altered proteins. Buelow *et al*. [39] have shown that certain C- and N-terminal truncations of SEB fused to protein A possess mitogenic activity. They also found, however, that the use of protein A resulted in significantly impaired fusion protein activity for both the full-length SEB and SEB truncations.

In the present report, we describe a characterization of the functional activity of N- and C-terminal truncations of SEB using the recombinant truncation fused to bacterial maltose-binding protein (MBP). In an effort to assess superantigen activity of truncated SEB and the full-length SEB as fusion proteins, we have measured mitogenic activity, MHC class II-binding activity, and TcR Vβ allele selectivity. Our results provide further information regarding the participation of both N-terminal and C-terminal residues in the superantigen activity of this toxin, and demonstrate that MBP fusions can be utilized to assess the functional activities of these microbial toxins.

## 2. Results and Discussion

### 2.1. Proliferative Responses of Murine Splenocytes to MBP-SEB Fusion Proteins

**Figure 1:**
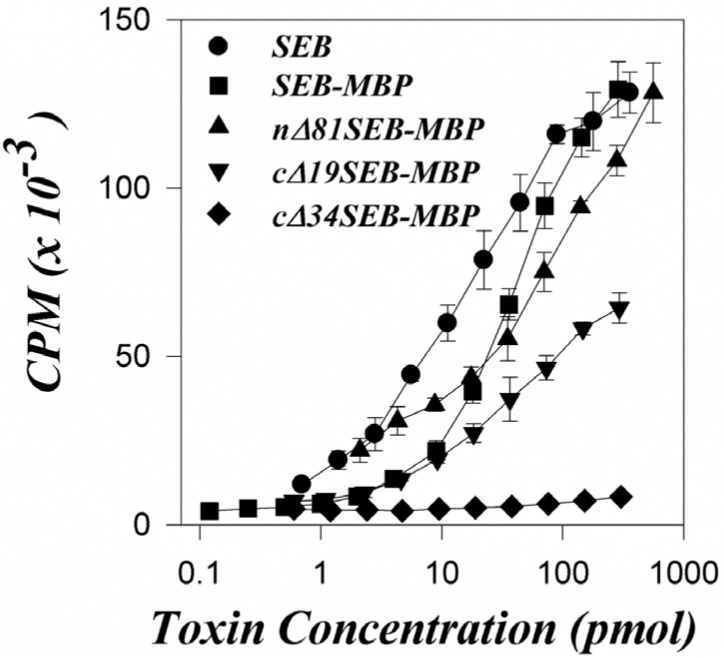
Proliferative response of murine C3H/HeJ splenocytes to staphylococcal enterotoxin B (SEB) or SEB-MBP fusion proteins. The proliferative response to various concentrations of SEB, SEB-MBP, nΔ81SEB-MBP, cΔ19SEB-MBP, and cΔ34SEB-MBP is shown. The response to MBP alone was not detectable (data not shown). Results show the mean of quadruplicate values ± standard deviation. The control responses (no mitogen added) were 6584 ± 890 cpm.

We compared the mitogenic activity of the fusion proteins with that of SEB. The results of a representative experiment (Figure 1) show that the full-length SEB fusion protein (SEB-MBP) induced a strong response, and the mitogenic activity of native full length SEB was approximately twice that of SEB-MBP (SEB ED_50_: 70 pmol; SEB-MBP ED_50_: 170 pmol). Surprisingly, the proliferative response induced by the truncated-SEB fusion proteins nΔ81SEB-MBP was also substantial, and was not significantly different from the full-length fusion protein (ED_50_ for nΔ81SEB-MBP: 180 pmol). In contrast, the C-terminal truncations exhibited significantly reduced mitogenic activity relative to the full-length fusion protein. The cΔ19SEB-MBP fusion induced a response which was less than 20% of that observed with the full-length fusion (ED_50_ for cΔ19SEB-MBP: 900 pmol). Control experiments show that MBP alone does not exhibit detectable mitogenic activity (data not shown). We have observed essentially identical results in experiments carried out with BALB/c and B10.BR mice. The results of these experiments suggest that the 81 amino-terminal and 19 carboxy-terminal amino acids of SEB are not mandatory for significant mitogenic activity. It is clear, however, that the potency of the cΔ19SEB-MBP fusion protein is greatly reduced relative to the full-length fusion protein or to native SEB alone. On the other hand, the deletion of 34 carboxy-terminal amino acids (cΔ34SEB-MBP) appears to eliminate virtually all of the mitogenic activity of the superantigen.

### 2.2. Analysis of Fusion Protein Binding to HLA Class II Antigens

**Figure 2:**
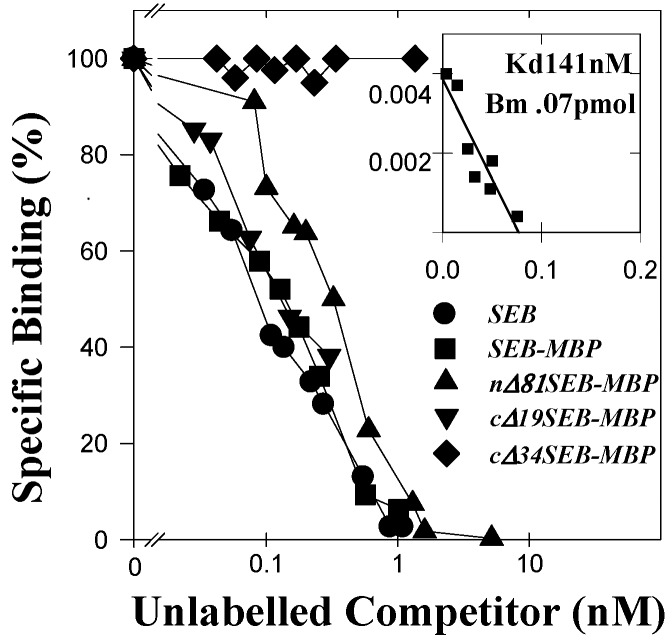
Analysis of fusion protein binding to HLA-DR1-bearing cells. Binding of radiolabelled SEB to DAP.3-DR1 was carried out in competition with unlabelled SEB, SEB-MBP, nΔ81SEB-MBP, cΔ19SEB-MBP, and cΔ34SEB-MBP. The insert shows a representative Scatchard analysis for binding of SEB to DAP.3-DR1 cells. The inserts represent plots of bound/free (ordinate) *vs*. bound (abscissa).

We attempted to analyze the HLA class II-binding characteristics of the MBP-SEB fusion proteins. Our experiments were carried out with fibroblasts transfected with HLA-DR1 (DAP.3-DR1). The results of binding experiments (Figure 2) show that these cells bind SEB with a dissociation constant (kd) of 141 nM. The Scatchard analysis is consistent with a binding density of 40,000 binding sites per cell. We then carried out an analysis of the capacity of each of the fusion proteins to compete with SEB for binding to the HLA-DR-bearing cell line. The results of a representative experiment show (Figure 2) that SEB-MBP and cΔ19SEB-MBP bind to the transfected fibroblasts in a manner which is essentially equivalent to that of SEB. The average concentrations of SEB-MBP and cΔ19SEB-MBP required to achieve 50% competition with SEB for binding to DAP.3-DR1 (determined from the mean of four experiments) are approximately 32 and 35 pmol, respectively, compared with about 25 pmol for SEB. The binding by nΔ81SEB-MBP appears to be somewhat weaker, and the concentration for 50% of binding of SEB to DAP.3-DR1 is 85 pmol. These results suggest that the binding affinities of SEB, SEB-MBP, and cΔ19SEB-MBP for HLA-DR1 are essentially equivalent. It is clear that the cΔ34SEB-MBP fusion protein fails to exhibit any detectable competition for the binding of SEB to these cells. In addition, control experiments have shown that non-transfected fibroblasts fail to exhibit detectable binding by SEB, and finally, the MBP carrier protein does not exhibit detectable competition for the binding of SEB to DAP.3-DR1 (data not shown).

### 2.3. Growth of Murine T Cells Following Stimulation with Fusion Proteins

It is well established that T cells stimulated with bacterial superantigens expand in a TcR Vβ-allele selective manner in the presence of IL-2 [42]. We attempted to characterize the Vβ-allele selectivity following stimulation with the MBP-SEB fusion proteins. Following three days of stimulation with SEB or the fusion proteins SEB-MBP, nΔ81SEB-MBP or cΔ19SEB-MBP, purified murine T cells were cultured for two days with interleukin-2 (IL-2), and the surface TcR Vβ-allele expression was determined. Our results (Table 1) show the expected expansion of cells bearing the responsive TcR Vβ 8.1 and 8.2 alleles following stimulation with either SEB or the fusion proteins. Responding cells which bear CD25 and Vβ8.1 and 8.2 increase from 26.7% to between 53.4% and 68.3% of the total population following SEB or fusion protein stimulation. T cells which bear the nonresponsive TcR Vβ6 allele are reduced from 8.7% in the concanavalin A (Con A) control group, to between 1.5% and 3.3% in the SEB and fusion protein groups. Analysis of cΔ34SEB-MBP or MBP alone was not included in these studies, because these agents are not mitogenic and do not yield detectable levels of CD25-bearing T cells at the termination of culture.

**Table 1:** Frequency of murine TcR Vβ alleles following stimulation of C3H/HeJ mice with Con A, SEB, SEB-MBP, nΔ81SEB-MBP, or cΔ19SEB-MBP. Results are expressed as the percentage of T cells co-expressing CD25 and the respective TcR Vβ allele, and are the means (±SEM) of four independent experiments.

Mitogen	% of Total T cells				
	Vβ6	Vβ8.1	Vβ8.2	Vβ8.3	Vβ7
Con A	9.3 ± 0.7	6.4 ± 1.0	20.6 ± 3.3	6.0 ± 0.4	10.7 ± 2.3
SEB	4.8 ± 1.6	11.0 ± 0.6	41.7 ± 4.0	20.9 ± 4.1	15.0 ± 1.2
SEB-MBP	3.9 ± 2.2	11.9 ± 0.6	42.8 ± 2.8	20.2 ± 4.3	14.6 ± 0.5
nΔ81SEB-MBP	3.0 ± 2.6	13.1 ± 0.9	44.4 ± 3.9	23.5 ± 3.8	16.1 ± 2.4
cΔ19SEB-MBP	3.9 ± 2.9	13.9 ± 0.8	49.5 ± 9.3	22.4 ± 3.7	17.0 ± 2.1

A variety of approaches have been utilized to identify the structural basis of bacterial superantigen activity. We have employed the recombinant truncation/deletion method in an effort to characterize the role of amino-terminal and carboxy-terminal amino acids in superantigen function. Our results strongly suggest that the amino-terminal 81 and carboxy-terminal 19 residues are not mandatory for substantial superantigen activity. These results may or may not be consistent with the experimental findings of some of the investigators who have examined the role of the amino-terminal region of the bacterial superantigens. Most noteworthy are the results of investigators who have generated mutant toxins by site-specific mutagenesis [20,21,22,23,24]. Using this approach, Kappler *et al*. [42] have identified three regions within the amino-terminal 61 amino acids of SEB which appeared to be involved in either the interaction with MHC class II or with the TcR. Harris *et al*. [22] generated mutant SEA toxins with amino acid substitutions at positions 25, 47, and 48 which failed to exhibit normal mitogenic activity. On the other hand, a number of investigators have shown by amino acid substitution analysis with SEA, SEB, SEE, SPEA and TSST-1 that numerous residues in both the amino- and carboxy-terminal regions of these toxins appear to be involved in superantigen function [20,22,23,26]. Based on the crystal structure of both SEB and TSST-1 [27,28], the residues identified by these investigators appear to reside on at least three separate faces of the superantigen.

The crystal structure data of the complex of SEB with HLA-DR1 [33] provides evidence that the both amino- and carboxy-terminal residues of SEB participate in the interaction with the MHC class II. These sites are composed primarily of residues in the regions 43–47, 65–78, 92–96 and 211–215. It is apparent from the crystal structure that roughly half of the residues of SEB which interact with class II are located in the amino-terminal 81 amino acids, and our studies with the truncation of these N-terminal amino acids retains substantial class II binding activity. However, we do observe a measureable reduction in class II binding activity with this truncation. On the other hand, our results suggest that the amino acids in the carboxy-terminal 158 residues contribute significantly to the class II interaction, and this is likely due to the contribution of the remaining class II-binding residues. Moreover, a comparison of our results with the two carboxy-terminal truncations shows substantial activity with the 1–220 region, but no detectable activity with the 1–205 region. This suggests that very critical MHC binding residues are located in the 206–220 region, and this would be fully in agreement with published crystal structure data showing MHC II contact sites in the 211–215 region [33].

The crystal structure of the complex of SEB with the TcR β-chain reveals contact sites on the SEB surface that are distributed over both N-terminal and C-terminal regions [34,35,36]. These contact residues include T18, G19, L20, E22, N23, N60, Y91, F177, E210, and L214. Our results here show that substantial mitogenic activity is retained following deletion of the N-terminal 81 amino acids, and this suggests that the loss of the TcR contact residues T18, G19, L20, E22, N23, and N60 are not mandatory for interaction with the TcR, both in terms of the proliferative response, and the TcR Vβ-allele specificity of the response. In contrast, the mitogenic activity of the C-terminal truncations is much more substantially reduced, suggesting that the contact sites in this region have more substantial TcR interaction activity. Analysis of the results with the carboxy-terminal truncations suggests that residues in the 221–239 region contribute substantially to the interaction with the TcR, since the proliferative response is clearly reduced with this truncation, yet the interaction of this truncation with MHC-II remains essentially intact. It should be appreciated that while the interaction of the 1–220 region with TcR may be reduced (based on reduced mitogenic activity), the Vβ-selectivity of this truncation remains unaltered. 

An additional approach to the question of the location of structural epitopes involved in superantigen function has been the generation of toxin chimeras. Results using SEA/SEE chimeras [25,26] have suggested that residues corresponding to residues 208 and 209 of SEB participate in TcR β-chain allele selectivity. The crystal structure of SEB shows that residues in this region form a part of the surface of one face of the toxin. The location of the TcR interaction site in the region which includes the carboxy-terminal residues is consistent with the results reported here. The mitogenic activity of the fusion protein cΔ19SEB-MBP is significantly reduced relative to full-length SEB, while the class II-binding activity of this fusion protein is essentially normal. These results suggest that the altered mitogenic activity of cΔ19SEB-MBP is due primarily to an altered ability to bind to the TcR.

We have examined the TcR Vβ-allele selectivity of the fusion proteins by fluorescence-activated cell sorter analysis. Our results show similar expansion of the responsive Vβ alleles in each of the fusion proteins when compared to the wild-type toxin. These results suggest that the reduced mitogenic activity of cΔ19SEB-MBP is not due to an apparent failure to selectively activate T cells. It should be pointed out, however, that analysis of this kind is not extremely sensitive to minor changes in selectivity, and it is possible that certain Vβ alleles may be more or less affected by loss of the 19 carboxy-terminal residues.

The superantigen truncation/MBP fusion protein approach described in these studies should have value in identifying regions of the superantigen responsible for toxin activities which are dependent on other binding interactions. For example, human colon carcinoma cells bind SEB in a class II-independent manner [43]. Moreover, we have previously identified a membrane protein expressed by a renal epithelial cell line, designated p85, which binds to SEB, SEC1, 2, and 3 [44,45]. Our previous studies, using this truncation/fusion protein approach, showed that the binding of SEB to p85 was dependent on residues in the C-terminal 19 amino acids since deletion of these residues eliminates all detectable binding activity. Our attempts to identify this protein have been unsuccessful at this point, and we have not been able to identify a membrane protein with this molecular mass which would be a likely candidate [44,45]. Indeed, we have previously eliminated the possibility that this protein is either MHC-II or MHC-I, and we established that SEB bound to p85 on epithelial cells does not allow for a productive interaction with the TcR on T cells [44,45]. The most likely interpretation of this result is that the complex of SEB with p85 does not result in access to critical TcR binding residues (most likely within the carboxy-terminal 19 amino acids). More recently, studies have shown that a dodecapeptide region of SEB (SEB152–161) is involved in the transcytosis of enterotoxins across intestinal epithelial cell monolayers [10]. The precise nature of the functional activity of this region is not clear at this time, but additional binding studies with epithelial cell populations may reveal more detailed information about the interaction of SEB with these cell types. We suggest that the deletion/MBP fusion method may be particularly useful as these studies progress. 

Taken together, the results suggest that the activation of T cells by SEB involves interactions between multiple cell populations. It is generally accepted that for superantigens like SEB and SEC, the binding of superantigen to the MHC-II expressed by antigen-presenting cells (predominantly dendritic cells and macrophages) occurs first, and this binding stabilizes and concentrates superantigen for presentation to the appropriate T cells [36]. Depending on the anatomical site, it is possible that epithelial cells in the environment may also bind (via p85) these superantigens, and essentially “block” productive interaction with T cells. However, for the antigen-presenting cells that bind SEB via MHC-II, the final step is for the complex of MHC-II-SEB to initiate a productive binding interaction with the TcR. This then leads to induction of TcR signaling cascades that can lead to T cell activation and proliferation in a TcR Vβ allele-specific manner. One could view the activation of T cells with SEB as a part of a dynamic interaction of T cells with SEB-bound to epithelial cells (non-productive for the T cell), and SEB-bound to antigen-presenting cells (productive for the T cell). This suggests that competition between epithelial cells and antigen-presenting cells for superantigen binding may dictate successful T cell activation and proliferation. 

## 3. Experimental Section

### 3.1. Bacterial Strains and Plasmids

Vectors used to generate the MBP fusion proteins, and for the expression of SEB, have been described in detail previously [45]. Briefly, the MBP fusion protein vector pMAL-C2 (New England Biolabs, Inc., Beverly, MA, USA) contains the *malE* gene upstream of a multiple cloning site, and the *entB* sequence, and truncations of the *entB* gene, were inserted to allow for the generation of MBP-fusion proteins with the carrier protein at the N-terminus of the inserted protein. The pMAL-C2 vector was engineered to remove the *malE* signal sequence in order to prevent the transport of MBP to the periplasmic space. 

### 3.2. SEB Constructs

The construction of MBP-SEB expression vectors carrying full length, and truncated, *entB* sequences, including the procedures for cloning and PCR amplification, have been described previously in detail [45]. The constructs utilized for the present studies were pTR6532 (full-length SEB), pTR65816 (N-terminal 81 amino acid truncation), pTR65192 (C-terminal 19 amino acid truncation), and pTR65342 (C-terminal 34 amino acid truncation).

### 3.3. Production of Fusion Proteins

Fusion proteins were prepared from cultures of transformed *E. coli* as previously described [45]. Briefly, bacterial cultures grown to mid-log phase were treated with 1 mM isopropyl-β-D-thiogalactoside, grown for an additional 3–4 h at 37 °C, and the bacteria were harvested and lysed with 25 mM Tris, pH 7.4, 10 mM EDTA, and 0.3% lysozyme. The treated bacteria were then subjected to rapid freeze-thaw, sonicated for 2 min, 0.5 M NaCl was added, followed by centrifugation at 10,000*g* for 30 min at 4 °C. The lysate was collected and purified by chromatography on an amylose column (New England Biolabs). Fusion proteins were eluted from the amylose column with 10 mM maltose. The purified proteins (>95% pure) were subjected to SDS-PAGE analysis and the identity of proteins was confirmed based on electrophoretic mobility and reactivity by western blot analysis (the relative molecular mass values: SEB-MBP 70.6 kDa, nΔ81SEB-MBP 62.5 kDa, cΔ19SEB-MBP 68.0 kDa, and cΔ34SEB-MBP 66.0 kDa). The western blot analysis was conducted using both polyclonal anti-MBP antibody (New England Biolabs, Beverly, MA, USA) and a monoclonal anti-SEB antibody, 2GD9, which recognizes a determinant in the C-terminal 140-amino acid region [18,46].

### 3.4. Proliferative Response Assay

The proliferative response of murine splenocytes to SEB and the MBP fusion proteins was carried out as described previously [15], using endotoxin-unresponsive C3H/HeJ splenocytes. Cultures of splenocytes (8 × 10^5^ cells in a volume of 0.2 mL in Dulbecco’s modified Eagle’s medium (DMEM) supplemented with 10% FCS and 50 μg mL^−1^ gentamicin). After 48 h, cultures were given 1 μCi of ^3^H-thymidine, and the cells were harvested after an additional 18 h. The proliferative response was assessed by measuring thymidine uptake. Mitogenic activity was assessed in part by determining the effective dose to elicit 50% of the maximal response (ED_50_).

### 3.5. Radiolabelled Cell-Binding Assay

The binding assay for the HLA-DR1-transfected murine DAP.3 clone DAP.3-DR1 [47] was carried out by a standard binding assay as described previously [48,49]. Briefly, the DAP.3-DR1 cells at a density of 2 × 10^6^ in 100 μL of cold competitor were diluted in a binding medium composed of DMEM containing 1% bovine serum albumin (BSA), 25 mM HEPES, and 0.05% azide. An additional 100 μL of ^125^I-SEB was added immediately, and the cells were incubated at 37 °C for 4 h. The cells were washed with binding medium, and then treated with 1 N NaOH. The radioactivity of the dissolved cells was determined with a gamma counter. SEB was radio-iodinated using the iodo-bead method as described previously [45]. A specific activity of 5–6 × 10^5^ DPM/pmol of SEB was normally achieved.

### 3.6. Flow Cytometry Analysis of Superantigen-Induced Murine T Cells

Using a minor modification of a standard protocol [7,49], murine T cells obtained from lymph nodes were purified by nylon wool and cultured at a density of 2 × 10^6^/mL with 4 × 10^6^ irradiated splenocytes and either Con A (10 μg mL^−1^), SEB (1 μg mL^−1^), MBP-SEB (2 μg mL^−1^), or nΔ81SEB-MBP (8 μg mL^−1^), or cΔ19SEB-MBP (10 μg mL^−1^) in DMEM supplemented with 0.1 mM non-essential amino acids, 1 mM sodium pyruvate, 50 μg mL^−1^ gentamicin, 2 mM l-glutamine, 10% FCS, 0.05 mM β-mercaptoethanol, and 10 μg mL^−1^ each of adenosine, uridine, cytosine and guanosine. The cells were harvested after 3 days, and viable T cells were returned to culture with IL-2 (25 U mL^−1^), cultured for an additional 2 days, and staining with antibodies for FACS analysis. Fluorescent antibody staining was carried out as described previously [50,51]. All monoclonal antibodies were obtained from Becton-Dickinson Laboratories (San Diego, CA, USA), and the cytometry was conducted using an EPICS Elite analyzer (Coulter Corporation, Hialeah, FL, USA).

## 4. Conclusions

We describe an approach to the localization of functional regions of superantigens in which truncations and/or deletions can be studied as fusion proteins using the maltose-binding protein. Our studies with SEB suggest that this approach allows for determination of T cell activation activity, as well as binding interactions with MHC class II or the alternative superantigen binding protein p85 since MBP does not possess functional activity on its own for either of these interactions. Finally, the studies presented here suggest that the superantigen functional activity of SEB is dominated by the contributions of residues in the C-terminal region of the protein.
